# The complete chloroplast genome sequence of *Begonia arachnoidea* (Begoniaceae)

**DOI:** 10.1080/23802359.2022.2087548

**Published:** 2022-06-23

**Authors:** Da-Yan Tao, Hai-Xia Yan, Jin-Ye Zhou, Jian-Bo Rong, Qian Song, Shi-Kai Guan, Shuming Luo

**Affiliations:** a Flower Research Institute, Guangxi Academy of Agricultural Sciences, Nanning, Guangxi, China; b Agricultural Science and Technology Information Research Institute, Guangxi Academy of Agricultural Sciences, Nanning, Guangxi, China; c Faculty of Sciences, School of Life and Environmental Sciences, Plant Breeding Institute, University of Sydney, Cobbitty, NSW, Australia

**Keywords:** *Begonia arachnoidea*, chloroplast, genome assembly, high-throughput sequencing

## Abstract

There are more than 2035 *Begonia* species (Begoniaceae) reported currently in the world. *Begonia arachnoidea* was found as a new species within a small area in Southern China. In this study, we are reporting for the first time its chloroplast genome for the purpose to compare with the chloroplast genomic data from other plant taxa which were closely related to this new species. Our results show that the circular chloroplast genome of *B. arachnoidea* is 169,725 bp in length, with 35.49% GC content. The whole structure of the genome has 76,431 bp in a large single-copy (LSC) region, 18,146 bp in a small single-copy (SSC) region, and the two inverted repeat (IRs) regions are both 37,574 bp. There are 90 protein-coding genes, 8 rRNA genes, and 42 tRNA genes encoded in this genome. Final phylogenetic analysis revealed that *B. arachnoidea* is genetically closest to *B. pulchrifolia* and *B. coptidifolia*.

*Begonia* L. (Begoniaceae) is one of the largest genera of flowering plants (Frodin 2004), and has 2,035 accepted species (Hughes et al. 2021). In China, more than 300 species have been estimated and new taxa remain to be discovered and described (Tian et al. 2018). After Yunnan Province, Guangxi is the second largest original center for *Begonia* species with 84 species (including infra-species) recognized (Dong and Liu 2018). Due to their asymmetrical, patterned, variegated foliage or attractive bright flowers, some *Begonia* species have been used as colorful indoor plants. Molecular phylogenetic analyses recently on *Begonia* still could not refine the boundaries for species lineages (Moonlight et al. 2019), and genomic variation of the chloroplast is significant for the classification of new species in this genus.

*Begonia arachnoidea* C.I. Peng, Y. Liu & S.M. Ku, 2008 has been reported in South China (Peng et al. 2008), which belong to *Begonia* sect. Coelocentrum Irmscher. This monoecious herb habitats on limestone walls in shrub or in mixed bamboo forest with an elevation of 200 meters, and occasionally on semi-overcast wet rocks. This species was named after its peltate leaves with venation patterns like a spider web. Currently, no phylogenetic studies have been carried out on this species. This study aims to report for the first time its complete chloroplast DNA sequence to provide a foundation for conservation, implementation and utilization, and subsequent species or infra-species classification.

In May 2020, leaf samples of *B. arachnoidea* were collected from Encheng, Daxin, Guangxi Province, China (22°44′04″ N; 107°07′01″ E). Plant sample was deposited in the nursery at the Flower Research Institute, Guangxi Academy of Agricultural Sciences (http://www.gxaas.net/), with a voucher number GXAAS-B00170 (collected by Jin-Ye Zhou, ahzhoujy@163.com). Total DNA was extracted using the CTAB method (Doyle and Doyle 1987) and sequenced on the Illumina NovaSeq 6000 platform from Annuoyouda Biotechnology Co., Ltd. (Zhejiang, China). Using a 150 bp paired-end sequencing strategy, 5.9 GB of high-quality clean reads were generated with adapters trimmed. Subsequently, the complete chloroplast genome was assembled by SPAdes v3.10.1 (Bankevich et al. 2012). Genome annotation was performed using CPGAVAS2 (Shi et al. 2019) and submitted to GenBank with an accession number MZ671994.

The assembled chloroplast of *B. arachnoidea* is a closed circular molecule with 169,725 bp. With an overall 35.49% GC content, the genome contains a large single-copy (LSC) region of 76,431 bp, a small single-copy (SSC) region of 18,146 bp, and two inverted repeats (IR) regions are both 37,574 bp. The complete chloroplast genome contained 140 genes, including 90 protein-coding genes, 8 rRNA genes, and 42 tRNA genes.

Complete chloroplast genomes of 21 other species in the Rosanae were selected for phylogenetic analysis. Species *Acacia dealbata* and *Bauhinia blakeana* were designated as the out-group. All the sequences were aligned using MAFFT v 7.429 (Katoh and Standley 2013) and trimmed by TrimAl (Capella-Gutierrez et al. 2009). The maximum-likelihood (ML) tree was constructed using bootstrapping with 1000 replicates through RAxML v8.0 (Stamatakis 2014) under the GTR + G model. The findings of the phylogenetic analysis suggested that *B. arachnoidea* is genetically closest to *B. pulchrifolia* and *B. coptidifolia*, and is closer to the genera *Lagenaria*, *Siraitia* and *Corynocarpus* in the Cucurbitales. Conversely, it is more distant to the *Acacia* and *Bauhinia* species in the Fabales (Figure 1).

**Figure 1.**
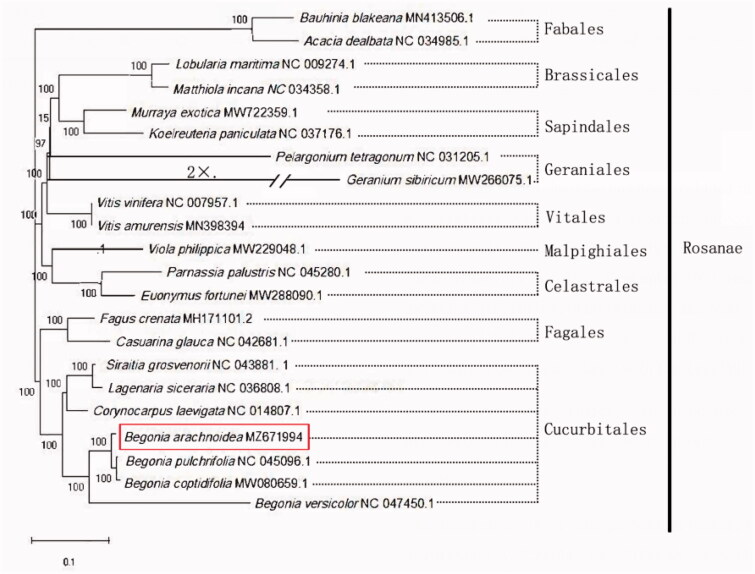
The ML phylogenetic tree based on the complete chloroplast genomes of *B. arachnoidea* and 21 other published species.

## Ethical approval

Even though *B. arachnoidea* has been included in the List of National Key Protected Wild Plants as a secondary protected plant by the National Forestry and Grassland Administration & Ministry of Agriculture and Rural Affairs in China since September 2021 (National Forestry and Grassland Administration & Ministry of Agriculture and Rural Affairs of the Peoplès Republic of China 2021), research activities including field studies, plant collection and related experimentation had been completed earlier in June 2021, all the work were carried out in accordance with the Regulations of PRC to do with wild plants protection. Therefore, it was not necessary to obtain specific permission or license for this research project.

## Data Availability

The genome sequence data for the accession number MZ671994 are openly available in GenBank of NCBI at https://www.ncbi.nlm.nih.gov/. Its associated BioProject, SRA, and Bio-Sample numbers are PRJNA770848, SRR16308659, and SAMN22241850, respectively.
